# Distinguishing participants, patients and the public: implications of different institutional settings on engagement approaches

**DOI:** 10.1186/s40900-025-00732-0

**Published:** 2025-07-15

**Authors:** Anne L. Buffardi, William Lammons, Nira Shah, Dalya Marks

**Affiliations:** 1 London Borough of Islington, London, UK; 2 Department of Primary Care and Population Health, University College London, Applied Research Collaboration (ARC) North Thames, London, UK; 3 Department of Public Health, Environments and Society, London School of Hygiene & Tropical Medicine, London, UK

**Keywords:** Public involvement, Engagement, Local authority, NHS, University, Resident, Patient, UK Standards for Public Involvement

## Abstract

**Background:**

There is an established history of patient and public involvement and engagement (PPIE) in academic and clinical research. As the National Institute for Health and Care Research (NIHR) expands its investment in research on and by local authorities (LAs), NIHR PPIE frameworks are increasingly being applied in this new context. This article examines if and how the relationship between the public varies across universities, the NHS and LA and what this means for PPIE.

**Methods:**

To analyse differences in institutional structures, we reviewed organisational websites, comparing the purpose and responsibilities of the institution, funding sources, governance structures, ability to directly action research findings, the role of public collaborators and duration of this relationship. We then systematically analysed these differences against the six UK Standards for Public Involvement: inclusive opportunities, working together, support and learning, governance, communications and impact. We also held a group discussion with nine PPIE Research Advisory Panel members to sense check if and how they perceived differences across these three institutional contexts and to refine and identify additional hypotheses about what might need to be adapted for PPIE in a LA setting.

**Results:**

The three institutions generally fall along a continuum, with universities having the most bounded relationship with the public and LAs the most expansive and enduring. The NHS and LAs have statutory responsibilities to the public, who finance their services and whose rights are articulated in institutional constitutions. Reflective of the service delivery responsibilities of both institutions, they are able to directly implement research findings, whereas university research outputs predominantly aim to inform others’ service design and delivery. Given these differences, our analysis suggests that the three standards on working together, governance and PPIE impact may require greater adaptation in LA settings. At the heart of the challenge is role clarification, since public contributors to research may also be council tenants, taxpayers and voters.

**Conclusions:**

PPIE in LA research offers new opportunities and challenges, requiring tailored guidance that accounts for the unique relationship between LAs and the public. We encourage PPIE contributors, coordinators and scholars across institutional settings to work together to fill this gap.

**Supplementary Information:**

The online version contains supplementary material available at 10.1186/s40900-025-00732-0.

## Abstract (plain language summary)

The National Institute for Health and Care Research (NIHR) is increasing funding for research on and by local authorities (LAs). However, recommendations on how researchers should involve the public were developed based on research at universities and the NHS. This article compares the relationship between the public and universities, the NHS and LA and what this means for public involvement.

We reviewed websites and documents and compared the differences of these three types of organisations against the six UK Standards for Public Involvement: inclusive opportunities, working together, support and learning, governance, communications and impact. We also discussed our questions with nine Research Advisory Panel members to see if and what they thought the differences were between universities, the NHS and LA and how public involvement approaches may need to be adapted for LA-led research.

The NHS and LAs have legal responsibilities to the public, who pay for their services and whose rights are listed in NHS and LA constitutions. These two types of organisations deliver services and so are able to directly take action based on research findings. In contrast, universities have a more clearly defined relationship with the public that is specific to research and rely on other organisations to implement their research findings. Given these differences, the three UK Standards for Public Involvement on working together, governance and impact may need to be changed somewhat, especially for LA settings. We encourage public contributors, coordinators and scholars across organisational settings to work together to fill this gap.

**Supplementary Information:**

The online version contains supplementary material available at 10.1186/s40900-025-00732-0.

## Background

There is an established history of patient and public involvement and engagement (PPIE) in research,^1^what scholars characterise as the ‘patient participation imperative’ [3]. In parallel, local authorities (LAs) often speak about residents being at the heart of their work, the importance of community voices in shaping strategy and services [4]. Moreover, the National Institute for Health and Care Research (NIHR) is expanding its investment in research on and by LAs, recognising the importance of the wider determinants of health as a key contributor to the health of a population. In addition to studies funded through the Public Health Research Programme and evaluations of LA interventions conducted by Public Health Intervention Responsive Studies Teams (PHIRST), 30 Health Determinants Research Collaborations (HDRCs) have been funded across the country to increase research capacity in LAs [5]. PPIE is a mandatory component of all these studies and initiatives. Therefore, NIHR PPIE frameworks developed in academic and clinical settings are increasingly being applied in LA contexts.

As these programmes are rolling out in practice, we take a step back to ask if and how the relationship between members of the public differs across university, NHS and LA settings and what this means for PPIE. This article aims to start disentangling what involvement of patients and publics entails, first by examining how relationships vary across these three institutional contexts and then analysing the implications of these differences for how public involvement is structured, implemented and measured. We are thus responding to de Graaff’s plea that it is ‘time to move beyond discussing PPIE as something that we can never have enough of and to start examining more thoroughly the work necessary to make PPIE work’ ([6], p.1903). Given the growing investment in research conducted by LAs, and relative under-exploration of the applicability of established PPIE frameworks to this setting, we place particular emphasis on PPIE in this context.

In the last decade, there has been a growing set of frameworks and guidance documents outlining how public contributors could and should be involved, with the aim of improving the quality and relevance of research by involving those who are directly affected by it [7]. Core guidance on PPIE, such as the UK Standards for Public Involvement [8], the Public Involvement in Research Impact Toolkit [9] and NIHR’s ‘different experiences’ framework for considering who might be involved in research [10], note the importance of taking a practical, flexible approach and adapting PPIE to different situations. None explicitly refer to specific institutional settings; however, the ten sites that formally piloted the UK Standards represented academic and clinical contexts [11, 12].

Guidance on ‘resident engagement’— the more commonly used term in LAs – tends to be oriented around statutory consultation requirements, which may involve hundreds or thousands of people [13, 14]. Resident engagement guidance also covers a much broader set of activities, often a version of the ladder of participation that ranges from informing to consulting, involving, co-production and more devolved resident-led decision-making [15], including types of interaction where residents are more passive *and* where residents have more power in decision-making. Thus, it includes what the academic and clinical communities characterise as PPIE, but also covers a broader continuum of involvement. Moreover, LA resident engagement has typically focused on design and delivery of government services rather than research. Therefore, there are differences in the scope and the focus of public involvement.

In terms of research on the design, practice and impact of PPIE, there is a paucity of studies or examples from practice in LAs compared to academic and clinical settings. A rapid literature search yielded very few articles on the topic, and to our knowledge, none focus specifically on PPIE *led* by LAs. For instance, of the 31 articles^2^ in this journal that include the terms ‘local authority’ or ‘local government’, 12 mentioned LAs as part of the background context, predominantly related to describing the study setting, but also the role of LAs in providing services, financial challenges and potential benefits of PPIE for LA [16–27]; 11 listed LAs among a broader set of stakeholders who were considered, planned to be or actually involved [12, 28–37]; 7 indicated they planned to or had shared results with LAs or mentioned the general implications for LA [38–44]; and one involved a review of LA housing strategies [45]. Elsewhere, LAs are similarly mentioned in relation to study settings, recruitment or as one of multiple stakeholders [46–50]. In all cases, the discussion about the role of LAs was very brief.

A recent review of 27 review articles assessing the impacts of PPIE also found very little on public involvement in LAs [51]. Notably, several of these studies reference the Local Government and Public Involvement in Health Act 2007 [52] as a landmark policy that formalised the use, need, and support for patient and public involvement in shaping health services, research, and other aspects of life within the domains of local government.^3^ The Act details the role of public involvement in LAs, and many PPIE scholars mark it as a policy turning point in which PPIE was seen as mandatory for creating and improving all public services, rather than it being a unique phenomenon of health research [57–63]. This perceived turning point over 15 years ago, however, has not been reflected in published literature on PPIE in LA research.

Thus, there is a gap in the evidence related to both processes and outcomes: how PPIE in research led by LAs is implemented in practice and what are the effects of different types of involvement across different LA contexts. As an initial step in advancing this emerging evidence base, we draw on existing resources – the UK Standards for Public Involvement: 1) inclusive opportunities, 2) working together, 3) support and learning, 4) governance, 5) communications, and 6) impact – and examine what, if anything, needs to be adapted for LA settings. We examine two interconnected questions: how does the relationship between researchers and the public vary across institutional contexts and what are the implications for PPIE of these different relationships? We aim for this paper to inform and inspire more substantive research and discussion on the topic among current and potential PPIE contributors, LA staff, academics who study PPIE and relevant funding agencies.

## Methods

To analyse differences in institutional structures across universities, the NHS and LAs, we undertook a review of organisational websites. We first identified sector-wide associations: Universities UK, the Higher Education Statistics Agency, Department of Health and Social Care, and the Local Government Association, as well as third-party organisations who describe organisational functions, such as the Institute for Government. When sector-wide information was not available – for example specific university governance processes and rights outlined in individual LA constitutions – we included examples from research active institutions who have received NIHR HDRC funding to strengthen LA research capacity and with which we are affiliated: University College London and Islington council.

We systematically searched these organisational websites and documents to identify four core elements of institutional structures: the purpose and responsibilities of the institution, primary funding sources, rights of the public as specified in constitutions, and governance structures or processes. Based on this information, we then characterised the duration of the relationship between the public and the institution and the organisation’s ability to directly action research findings. This approach, therefore, identifies formal aspects of these three institutional types, rather than informal norms [73].

To analyse the implications of these institutional relationships, we systematically compared data from Table 1 against the six UK Standards for Public Involvement to identify the implications for each of the three settings: universities, the NHS and LAs. These six standards were developed by the UK Public Involvement Standards Development Partnership, with representatives from the Chief Scientist Office in Scotland, Health and Care Research Wales, the Public Health Agency in Northern Ireland and the National Institute for Health and Care Research in England. From 2016–2018, the standards were developed and refined through an initial literature review, a series of meetings and workshops, a public consultation and pilot testing in a range of projects [11, 12]. The categories and terminology we use here reflects the wording of the standards: inclusive opportunities, working together, support and learning, governance, communications and impact.

Finally, we held a 1.5-h online group discussion with nine members of the NIHR Applied Research Collaboration North Thames Research Advisory Panel (RAP) [74], all of whom have firsthand PPIE experience with universities, NHS and/or LAs. The aim of this group discussion was to sense check if and how these panel members perceived differences across these three institutional categories and to refine and identify additional hypotheses about what might need to be adapted for PPIE in a LA setting. This discussion was recorded, with verbal consent from contributors, and transcribed. We conducted thematic analysis of responses to each group discussion question, noting which themes and UK Standards for Public Involvement were more salient and when there was consensus or differences of opinion among panel members.

## Results

### How does the relationship between researchers and the public vary across institutional setting?

Across the categories along which we analysed institutional differences, universities, the NHS and LAs generally fall along a continuum: universities having the most bounded relationship with the public and LAs the most expansive and enduring, with the NHS falling between the two Table 1.

**Table 1:** Institutional orientation and nature of the relationship with the public across university, NHS and local authority contexts

	**University**	**NHS**	**Local authority**^**a**^
*Purpose & responsibilities of the institution*	Teaching, scholarship & research [64]	Improve, prevent, diagnose & treat physical and mental health problems [65]	Over 800 services, categorised into 33 broad areas including social care, aspects of transport, housing, education and health, libraries and waste collection [66–69]
*Primary funding sources*	Tuition fees Research grants Central government grants [70]	National taxation [65]	Central government grants Council tax Business rate revenue [66]
*Role(s) of the individual in relation to the institution*	PPIE contributor Student	PPIE contributor Patient Rights outlined in the NHS constitution [65]	PPIE contributor Resident Direct taxpayer Voter Service user / Tenant Rights outlined in council constitutions [71]
*Governance structures, including additional institutional mechanisms for public involvement beyond PPIE in research*	Example: UCL Council (appointed and elected academic & student members), Academic Board, University Management Committee [72] Limited additional mechanisms for public involvement	Central government sets the framework for the NHS, accountable to Parliament for its operation, organisation of services by local Integrated Care Systems [65] Additional public involvement through complaints mechanisms [65]	Councillors elected on 4-year terms [66] Additional public involvement through councillor advice surgeries, public sessions of full Council meetings, statutory consultations, complaints mechanisms [71]
*Ability to directly action research findings*	Low	Medium to high, within financial constraints	Medium, within financial and regulatory constraints
*Duration of the relationship*	Time-bound	Enduring	Enduring

^a^Here we focus on local government in England and single-tier rather than double-tier areas, where responsibilities are shared by county and district councils

In terms of thematic scope, the Local Government Association estimates that LAs are responsible for over 800 services from social care to waste collection, including aspects of health (e.g. sexual health and smoking cessation services), whereas the NHS focuses exclusively on health. Thus, the public may be interacting with LAs on a wide range of issues, predominantly related to services rather than research, and the range of topics on which LAs could conduct research varies widely. The research topics with which universities engage the public may be similarly diverse, but the core focus of the engagement is research. And, in terms of both the services they provide and research they conduct, the NHS is more thematically bounded, focused solely on health.

The public indirectly finance all three institutions through taxes collected through various means, but LAs are the only institution that directly bills households through council tax collection. Therefore, the public’s financial contribution to LAs might feel more visible to residents than, for example, value added tax (VAT) on purchases, or National Insurance and income tax, even though the proportion of council tax to income is substantially lower relative to tax contributions to central government.

Indeed, residents have several relationships with their LA. They are direct council taxpayers, registered voters, use local services such as libraries or waste collection, and some may be LA tenants – all alongside being a PPIE contributor. In addition to engaging in PPIE processes related to research, there are multiple institutional mechanisms through which the public can be involved and input into LA decision-making processes. This takes place most directly by voting, and more regularly through councillor advice surgeries, public sessions at full council meetings and statutory consultations.

In the NHS, PPIE contributors are also patients, a more defined relationship. Within LA and NHS settings, these relationships are enduring; the public are patients and citizens or residents over time. In contrast, the public’s relationship with universities is more likely to be defined by their specific PPIE research role, often time and issue-bound by a research study or programme. That said, research networks and infrastructure programmes are increasingly establishing standing PPIE panels (such as the RAP), which enable longer-term relationships.

Both LAs and the NHS have statutory responsibilities to the public, whose rights are articulated in institutional constitutions. This is not the case for universities. The public therefore has a voluntary relationship with universities and can chose whether or not to engage or affiliate with them. In contrast, by simply residing in a particular geographic area and in the UK overall, the public automatically has a relationship with their local authority and the NHS and cannot, for instance, opt out of paying taxes to finance these services.

Reflective of the service delivery responsibilities of both the NHS and LAs, they have the potential to be able to directly implement research findings. Universities, on the other hand, provide the research outputs to others to support their service design and delivery. Universities do not have to fulfil statutory duties to the public, and as such, are dependent on other institutions being aware of, accepting, financing and implementing their research results.

We note, however, that the opportunity for LAs to action research findings is limited by considerable financial and regulatory constraints. LA core spending power has been declining over the last 15 years [75, 76] and the scope of LA implementation in many areas is bounded by national regulations and guidance, what Jones and Stewart characterise as a dominant centralist approach [77].

We now examine the implications of these differences for PPIE.

### What are the implications of differences in these relationships for PPIE?

The UK Standards for Public Involvement cover six domains: working together, governance, impact, communications, inclusive opportunities and support and learning – the first three which map onto Table 1: roles, governance and actioning findings. In Table 2 we assess the implications of the different relationship between the public and universities, the NHS and LAs across the six standards. We present the standards in order of prominence, where the differences appear to be the greatest for working together and governance, less so for support and learning.

**Table 2:** Implications of UK Standards for Public Involvement across university, NHS and local authority contexts

UK Standards for Public Involvement	University	NHS	Local authority
**Working together** in a way that values all contributions and that builds and sustains mutually respectful and productive relationships *Is there a shared understanding of roles, responsibilities and expectations of public involvement?*	• Clearest roles and responsibilities • Shorter-term relationship explicitly linked to a research project	• Potential for role confusion between patient and research contributor, less so if the study is conducted by a different entity (e.g. clinical research centre) than a regular health care provider	• Greatest potential for role confusion due to overlapping relationships as a PPIE contributor, service user, tenant, voter, taxpayer and therefore, potentially higher expectations that public involvement will lead to direct changes • Potential misunderstanding of the difference between what is mandatory, strongly encouraged and voluntary, concerns about repercussions on access to services if contributions are perceived to be critical • Opportunity to develop longer-term, meaningful relationships
**Governance**: involve the public in research management, regulation, leadership and decision making *Are public voices heard, valued and respected in decision making?*	• Well-established, formalized research governance processes (e.g. Institutional Review Boards) • Limited involvement in defining research questions and methods, decisions regarding research management and regulation	• Well-established, formalized research governance processes (e.g. Institutional Review Boards) • Additional channels for public voice: complaints processes, Healthwatch advocacy	• Greatest range of channels for public voice (e.g. voting, direct communication with elected representatives, statutory consultations, complaints processes) • Potential confusion between decision-making about research (prioritising research topics) and broader council decision-making processes (extent to which findings influence policy)
**Impact**: seek improvement by identifying and sharing the difference that public involvement makes to research *Are the changes, benefits and learning resulting from public involvement acted on?*	• Potential to immediately, directly benefit research participants if offered investigational interventions • Scale up dependent on other institutions	• Potential to immediately, directly benefit research participants if offered investigational interventions • Greater control over scale up (within resource constraints) • Potential confusion between research study and services	• Greater control over scale up (within resource constraints) • Changes identified by the public may not be in LA control; lack of understanding of sphere of influence, confusion between central government and LA jurisdiction, research study and services
**Communications**: use plain language for well-timed and relevant communications as part of involvement plans and activities *Are the needs of different people being met through inclusive and flexible communication methods?*	Emphasis on academic audiences, potentially missed opportunities to share findings with practitioners and decision-makers if not proactively done or required	Emphasis on clinical audiences, potentially missed opportunities to share findings with practitioners and decision-makers outside of the NHS if not proactively done or required	• Emphasis on internal LA audiences, potentially missed opportunities to share findings with other LAs and sectors • Wide range of communications materials and channels, including council tax notices, so potential for study-related involvement opportunities and findings to be overlooked and/or confused with other messages, what is mandatory (e.g. taxes, rules in housing estates), strongly encouraged (e.g. voting) and optional (e.g. contributing to research) • More channels for recruitment and discussion of findings (e.g. electronic noticeboards in council housing)
**Inclusive opportunities** that are accessible and that reach people and groups according to research needs *Are people affected by and interested in the research involved?*	• More targeted opportunities to address specific research questions, typically with broader geographic reach • Potential for multiple requests from the same institution because of broader thematic scope (e.g. faculties of public health and environment)	• More targeted opportunities linked to specific conditions	• Opportunities for the entire borough population, current service users and specific subgroups, covering a wide range of topics • Potential for multiple requests from the same institution because of broader thematic scope (e.g. public health and environment departments)
**Support and learning**: opportunities that build confidence and skills for public involvement in research *Have specific resources been designated to support learning and development opportunities for both the public, researchers, and staff?*	• Established research training programmes, including public involvement training • Can signpost to external services who can support specific needs (not related to research), limited scope to directly address needs	• Opportunity for contributors to more directly link with other health practitioners to support other health needs	• Established training and council employment pathways, although comparatively few roles explicitly related to research • Opportunity for contributors to more directly link with other council staff (e.g. housing officers) who could provide support to address some needs

Each subsection is framed by the questions posed in the UK Standards for Public Involvement guidance.

#### Working together: is there is a shared understanding of roles, responsibilities and expectations of public involvement?

Given the multiplicity of roles individual members of the public have with LAs, there is greater potential for role confusion among residents, compared to their relationship with universities, which is more clearly defined. At the same time, LAs and the NHS offer greater opportunity to establish long-term relationships with the public than university-based research, as they provide opportunities to develop more meaningful and productive relationships. Therefore, establishing a shared understanding of roles, responsibilities and expectations of public involvement may require more discussion and clarification upfront in these settings and on an ongoing basis than for many university-based PPIE activities. This includes distinguishing between PPIE in LA-led research from statutory consultation, as well as being transparent about the scope or potential of PPIE to influence LA decisions related to service delivery.

Clarifying roles may be insufficient to overcome some of the negative effects of PPIE for both public contributors and researchers, including frictions or disagreements, tokenization, feelings of disempowerment, power imbalances, time and financial demands, and researchers’ difficulty in implementing PPIE contributors’ suggestions [58, 78–83]. Indeed, across all three institutional settings, there are inherent power imbalances between PPIE contributions and university, NHS and LA staff that this standard seeks to mitigate. However, given LA and NHS statutory responsibilities, public contributors may potentially have greater concerns about repercussions on their access to services if their contributions are perceived to be critical.

#### Governance: Are public voices heard, valued and respected in decision making? 

There are more mechanisms for public voice to be heard in NHS and LA settings, from statutory consultations and complaints mechanisms to proactive, potentially positive interactions with elected council members.

Universities and the NHS (unlike LA settings) have well-established *research* governance processes, including central research offices, Institutional Review Boards, Research Advisory Boards and Community Advisory Boards. Therefore, compared to LAs it may be easier and clearer both for PPIE contributors and staff to navigate how the public can participate in research governance processes.

However, because of the pre-determined focus of many research funding calls and the weight placed on professional specialisation in review criteria, there may be limitations in the extent to which public contributors can set the research agenda. Public contributors may be more involved in supporting specific aspects of the research, for example contributing to the development of data collection tools, rather than defining research questions or proposing specialised methods. Research ethics processes may be an area where the public can offer a unique contribution across all institutional settings. This is a role in which they are currently underutilised, as there are few examples of non-academics serving as permanent or mandatory members on ethics review panels in universities, the NHS or LAs.

#### Impact: are the changes, benefits and learning resulting from public involvement acted on?

The impact standard focuses on the difference that public involvement makes to *research*. In principle, the difference that public involvement makes to research should be similar across institutions. In terms of impacts on *services*, universities and the NHS may be more likely to be researching investigational interventions so may be able to offer public contributors who are also research participants immediate benefits if these interventions are effective, for example, access to experimental treatments or new programmes and care pathways.

For public contributors who aspire for their involvement in research to lead to changes in policy and practice, LAs and NHS are better placed to be able to directly implement research findings. PPIE members – or at least residents taking part in LA engagement activities – may be more motivated by the possibility of affecting impacts on downstream services that would directly lead to improvements to their lives. PPIE may influence LA research but not necessarily lead to changes in policies or services if these changes are beyond LA control (e.g. rent and house prices, mortgage rates) or are not feasible to implement within current resources (e.g. comprehensive housing retrofits to reduce carbon emissions).

In contrast, it is clear that implementing or scaling up research findings is beyond the scope of academic research. Public contributors may therefore overestimate what LAs are able to change, relative to their expectations of LAs versus universities. These expectations about the impact that their input can have on services may in turn lead to greater frustration when changes do not materialise.

#### Communication: are the needs of different people being met through inclusive and flexible communication methods?

The guidance related to the communication standard – plain language, well-timed, with an inclusive and flexible approach – is relevant for all three institutional settings. Each have their own core audiences (academics, clinical staff, elected members and council officers) whose information needs and communication styles might be different to the general public. Therefore, universities, the NHS and LAs would all need to tailor communication to a diverse range of public audiences. LAs communicate with residents about a wider range of topics, including council tax notices, local amenities and support services over the life course, so communication specifically related to PPIE contributions and research findings may be less noticeable.

#### Inclusive opportunities: are people affected by and interested in the research involved?

Across the three institutional settings, identifying who is affected by and interested in the research may be the most straightforward for the NHS, given their more bounded focus on health. Universities have both the broadest thematic and geographical scope. The scope for involvement is geographically bounded for LAs but could include the entire borough or city. In terms of contacting the public about opportunities to be involved, when more people are interested in being involved than are able to participate, establishing transparent selection criteria is important regardless of the institutional context. Compared with universities but similar to the NHS, it may be easier for LAs to contact existing service users or all borough residents through routinely held data records and to share PPIE opportunities through existing communication channels such as newsletters and e-bulletins.

#### Support and learning: Have specific resources been designated to support learning and development opportunities for both the public, researchers, and staff?

Of the six standards, the implications of the differences across institutional types appear the least marked for support and learning, or at least the challenges may be easier to overcome. Although each type of institution may be better positioned to provide specific types of support, they can all access external training and supplementary support services. For example, universities have established research training programmes and so may be best placed to directly build these technical skills. LAs, on the other hand, may be in a better position to link public contributors to services who can address specific needs. If the public is involved in a study about the effects of housing quality on health and perceived wellbeing, for instance, university researchers will have existing training modules on evidence appraisal and data analysis, whereas LAs may be able to directly connect PPIE contributors and research participants to council housing officers. Universities have established employment pathways for research; however, in recruitment LAs may place a higher value on community research or PPIE experience relative to academic qualifications.

#### Research advisory group reflections

The Research Advisory Group considered the implications of the differences between institutions reported in this paper; and, identified areas for future research and discussion about adapting existing PPIE guidance for use in LA settings, for instance, how PPIE contributors perceive their role and their expectations of PPIE impact. This initial brainstorming session with RAP members was more focused on differences between universities and LAs rather than the NHS, given the affiliation of the group and orientation of this paper.

RAP members observed the longstanding history of academic research and more established, formal research governance and PPIE processes, including ethics review boards, NIHR guidelines on PPIE and PPIE coordinator roles. Across the six standards, they spoke more about the communication, working together and inclusive research standards than about differences in impact or support and learning. Overall, RAP members expressed more positive PPIE experiences with academia and had more critiques on the ways in which LAs engaged with residents (not necessarily specific to research). Compared to LA processes, RAP members perceived universities to have more established feedback and communication mechanisms, greater awareness of the importance of diversity in PPIE and less pronounced power imbalances in terms of how staff interacted with PPIE contributors. They also noted differences in financial compensation, with universities consistently adhering to suggested NIHR hourly rates.

While there was broad agreement among the group on these points, the extent to which RAP members felt PPIE processes were institutionalised within universities and that LAs were committed to public engagement varied, based on the length and type of involvement different members had with these two types of organisations.

These initial RAP member observations underscore the importance of covering both relational and practical considerations when discussing how PPIE guidance can be tailored to LA settings. The latter may be more straightforward to shift, improving feedback loops for example, while LA staff and residents work through more challenging relationship dynamics over time.

## Discussion

This review documents differences across universities, the NHS and LAs in their purpose, funding sources, governance structures, type and duration of relationships with members of the public and ability to directly action research findings. Other differences in PPIE across the three settings – notably the lack of formalised *research* governance processes in LAs – may be more related to the nascent stage of LA-led research and correspondingly, public engagement in research.

The relationship between the public and universities is arguably the simplest, with clear boundaries which are defined by each research study. The NHS and LAs have legal responsibilities to the public, who finance their services. The relationship between the public and LAs is the most direct in terms of governance (voting) and financing (council tax). It may also be the most complex, given the range of roles and the varied nature of services and potential research topics LAs cover. Indeed, PPIE contributions to research may be a very small aspect of the public’s relationship with LAs. Thus, there are clear differences in the relationship between the public and these three institutions.

With LAs, there is greater potential that residents may link PPIE in research and statutory responsibilities to deliver services, which may lead to a greater gap between their expectations and outcomes. However, if appropriately structured and communicated, PPIE in LA-led research may lead to more actionable findings and longer-term relationships than in other institutional settings. Given their publicly funded statutory responsibilities, LAs may have more similarities with the NHS and may benefit from learning from their PPIE approaches, particularly related to role clarification and scope of impact. As more attention and resources become focused on the relationship between social care and health and transitions between home, community-based and secondary care, there may also be growing opportunities for joint research and PPIE initiatives. Moreover, with much of the recent funding for LA research coming from NIHR, local authorities could also learn from universities’ longstanding experience applying NIHR PPIE guidance.

There are clear interconnections across the six UK Standards for Public Involvement (working together, governance, PPIE impact, communication, inclusive opportunities, and support and learning). For instance, potential confusion over roles may affect expectations regarding the scope of impact of PPIE on policies and practices. Improved communication should help to clarify roles and enable more inclusive opportunities to be involved. Having formal research governance processes will provide a structure for regular communication, inclusive recruitment, support and learning. Therefore, improving one standard should have positive spillover effects on other standards.

That said, all of the statements above are hypotheses in need of testing. As noted at the outset, this article aims to characterise an evidence gap, pose questions and provide an initial analysis to identify plausible hypotheses to guide a future research agenda in this area. We call on PPIE contributors, coordinators and scholars across institutional settings to join in a broader national discussion about the implications of LA relationships for PPIE. Our initial discussion with RAP members represents an initial step that can now be deepened and expanded, both geographically and institutionally. Ideally, a larger national dialogue would lead to LA-tailored guidance, building upon our analyses and lessons from academia and particularly the NHS, as well as LAs’ own history of engagement and co-production related to service delivery.

Refined guidance should both inform and be informed by implementation research, comparative analyses of how PPIE is applied within and across different institutional settings: in different LA contexts and different types of studies, as well as between LA, NHS and university contexts. The field would benefit from a more nuanced understanding of how researchers and PPIE contributors navigate the opportunities and constraints of different settings, drawing on theories of organisational behaviour and the consideration of power dynamics discussed in participatory action research scholarship. Future studies should cover both processes and outcomes of specific PPIE approaches, intended and unintended, positive and negative.

Our analysis of institutional differences has focused on formal rules and processes, but informal norms and organisational ways of working may also be influential factors in shaping PPIE across different types of institutions. How universities, the NHS and LAs define what constitutes research, the purposes for which it is conducted and how, may in turn affect how PPIE is conceived and applied. Moreover, universities, the NHS and LAs are situated within broader systems, which also influence their research agendas and scope for action. Future research should therefore consider both internal and external factors shaping PPIE, including the direct and indirect influence of these three institutional types on one another.

As LA-led research matures, so too must PPIE.

## Supplementary Information

Supplementary Material 1.

## Data Availability

No datasets were generated or analysed during the current study.

## Abbreviations

HDRC: Health Determinants Research Collaboration
LA: Local authority
NIHR: National Institute for Health and Care Research
PHIRST: Public Health Intervention Responsive Studies Teams
PPIE: Patient and Public Involvement and Engagement
RAP: Research Advisory Panel
VAT: Value added tax

## Footnotes

1 NIHR acknowledges “The term research means different things to different people, but is essentially about finding out new knowledge that could lead to changes to treatments, policies or care. The definition used by the Department of Health is: ‘The attempt to derive generalisable new knowledge by addressing clearly defined questions with systematic and rigorous methods.’” [1]. While the Health Research Authority distinguishes between research, service evaluation, clinical/non-financial audit and health surveillance for the purpose of determining what requires NHS Research Ethics Committee review [2], what is considered research in a local authority context is very much a live debate. At the time of writing, the NIHR Research Support Service is conducting a Delphi study with LA participants from across the country, with the aim of reaching a shared understanding of the term. For the purpose of this article, which is focused on institutional differences in PPIE, rather than differences in the conceptualisation and application of research, we use the term research broadly, covering what universities, the NHS and local authorities currently consider as research.

2 These search terms returned 38 articles, three of which were duplicates and four of which did not mention local authority or local government in the article itself.

3 Arguably, the mandate that LAs consult with residents and partner organisations was codified eight years prior in the Local Government Act 1999 [53, 54], with foundations over a century before in the Local Government Act 1888, which established elected county councils [55, 56].
