# Supplementary material for: Distinguishing participants, patients and the public: implications of different institutional settings on engagement approaches
Source: Res Involv Engagem. 2025 Jul 15;11:84. doi: 10.1186/s40900-025-00732-0 (PMC12265212; doi:10.1186/s40900-025-00732-0)
Supplement: Supplementary file 1 — Supplementary Material 1. [file 40900_2025_732_MOESM1_ESM.docx]

**Distinguishing participants, patients and the public:** **Implications of different institutional settings on engagement approaches**

GRIPP2 Short Form

| **Section and topic** | **Item** | **Reported on page No** |
| --- | --- | --- |
| 1: Aim | Report the aim of PPI in the study | 6 |
| 2: Methods | Provide a clear description of the methods used for PPI in the study | 6 |
| 3: Study results | Outcomes—Report the results of PPI in the study, including both positive and negative outcomes | 12 |
| 4: Discussion and conclusions | Outcomes—Comment on the extent to which PPI influenced the study overall. Describe positive and negative effects | 12,13 |
| 5: Reflections/critical perspective | Comment critically on the study, reflecting on the things that went well and those that did not, so others can learn from this experience | 12 |

Please also note that one of the co-authors is a PPIE contributor (NS), so her inputs are reflected throughout the manuscript.
